# Courting emissions: climate adjudication and South Africa’s youth

**DOI:** 10.1186/s13705-021-00320-6

**Published:** 2021-11-22

**Authors:** Bright Nkrumah

**Affiliations:** Center for Human Rights, University of the Free State, Bloemfontein, South Africa

**Keywords:** Climate litigation, Rights, South Africa, Ubuntu, Youth

## Abstract

**Background:**

The urgency to pursue sustainable consumption or use energy in a manner that does not negatively impact the environment has become an important theme in recent times. As a major fluctuation in the atmosphere, climate change will be one of the major challenges faced by youth. As a result, there have been a growing number of young South Africans advocating for environmental justice. Surprisingly, their effort has not yielded the expected result as the country continues to emit a high amount of greenhouse gases. The notion of youth may be construed as those between the ages of 15 and 24. The age bracket suggests that the adult lives of this population will be shaped by environmental crises such as famines, vector-borne diseases, and hikes in commodity prices which may impinge on their basic rights to life, health, and property. This development triggers an ancient discourse, what role can youth play towards decarbonization? In other words, which effective avenue could be used by young people for capping emissions?

**Methods:**

An analysis of South Africa’s energy policy documents relevant to sustainability was conducted through the application of desktop research. We use (inter)national instruments and jurisprudence to understand how a state structure, like the judiciary, could nudge the executive to cap rising green gas emissions. South Africa is used as a case study because of its over-reliance on coal for electricity, and how young people could use the existing legal framework to cap rising emissions. Drawing from existing literature, the paper interrogates the lack of activism around climate litigation and under what conditions this pattern could be reversed in South Africa.

**Results:**

The paper found that while litigation has an important role to play in mitigating climate change, it ought to be complemented with other forms of advocacy.

**Conclusions:**

The study concludes that given the government’s perceived slow steps towards shifting from coal to renewables, youth (who will bear the brunt of high emissions) ought to use both courtrooms and advocacy to trigger political action.

## Background

Adapting to cold might be easy, but the same could not be said of hot climates. With simple acts of exercising, appropriate clothes, or huddling together, one will potentially overcome shivering. Yet, heat acclimatization requires cooling devices to enhance ventilation and heat loss [1]. This implies that individuals without the capacity to afford basic technologies such as fans or air-conditioning will be hit hard [2]. With human-induced greenhouse gas emissions (GHGs) increasing global warming, efforts towards mitigating these emissions are essential [3, 4]. This is more disconcerting as climate modelers predict extreme temperatures and heatwaves over the next three decades [5–8]. The time frame suggests that youth are more likely to bear the full brunt of a warming climate that is marked by stronger hurricanes, drought, and heatwaves [9].

As a major fluctuation in the atmosphere, climate change will be one of the major challenges faced by youth [10]. The notion of ‘youth'’ encompasses persons between the ages of 15 and 24 [11]. In South Africa, approximately 9.5 million of the country’s demography fall into this camp [12]. Indeed, there is an evolving number of young South Africans who have joined global campaigns, and often used print and electronic media to advocate for urgent climate action [9, 10]. One caveat bears underscoring, nonetheless. These social activists have arguably not triggered an overarching government response as the country’s GHGs grew has grown by 44% from 1990 to 2012 [13].

Thus, whereas the Paris Agreement imposes a duty on all state parties, regardless of one’s resources to frame and operationalize measures towards sustainability, compliance with this obligation has been fraught with delays and inaction [14]. To this end, there has been a growing number of citizens who seek to use courtrooms as avenues to nudge their government to respond to the threat of rising emissions [15–18].

Ironically, while a number of these litigants at the global level are youth, the agency and voices of young South Africans have rarely been articulated in courtrooms. This trend is even more disconcerting as scholarship around South Africa’s climate justice has had limited interaction with the role of local youth in equally invoking the Constitution and other legal instruments to trigger political action towards carbon neutrality [9, 10, 19]. The paper fills this gap by suggesting ways in which disempowered youth could use courtrooms to reclaim their rights to a healthy environment. It is expected that concerted climate action through litigation could stimulate strategies towards net-zero emissions by 2050 [9]. To complement the effort of young people in this regard, the paper sets out careful arguments which could be advanced in courtrooms, as well as feasible strategies which could be used to counter conceptual and practical constraints which might arise. It is anticipated that a combination of legal strategies and collective action from young people is likely to nudge the state towards shifting to renewables, or alternative energy sources which might have minimal ecological ramifications.

The paper is structured as follows. Excluding the present introduction, it is split into four sections. The next section begins with a brief description of the methodological approach used in conducting this research. The section then proceeds to provide an overview of the current state of climate change and its impact on young South Africans. Since a considerable number of these youth engage in external activities that expose them to heat, the section will infuse an assessment of heat on the health of this subpopulation. This assessment will be conducted in light of evolving heat stress in classrooms, poorly ventilated shacks, and young hawkers on hot pavements. The section proceeds to consider the extent to which the Constitution safeguards this subgroup from climate-related health hazards, such as heat stress. The assessment interrogates the inability of the state to translate the lofty ideals of section 24 of the Constitution into reality. In seeking to understand the protection afforded youth from environmental hazards, specific attention will be paid to this provision as it overtly entrenches the right to a clean environment. This assessment will be complemented with critical analysis of recent instruments, Climate Change Bill (CCB), National Environment Management: Air Quality Act (NEMA), and Carbon Tax Act (CTA), all aspiring to contribute to clean energy [10–12]. These documents will be assessed in light of their effectiveness in combatting emissions, and the leverage provided for young people to be included in framing and operationalizing climate actions at the local/provincial/national levels.

The third section considers the prospect of using courtrooms to ensure compliance with section 24 of the Constitution, particularly cutting down emissions from the energy sector. To achieve this end, it will draw from (inter)national jurisprudence to determine which claims could be advanced, and why. The final section summarizes the findings of the paper.

## Methods

The general methodological approach for this study reflects a policy analysis of low-carbon electricity pathways for South Africa, drawing from empirical surveys and international jurisprudence. We conducted (a) a literature and document review and (b) an analysis of local and international climate jurisprudence. The documents review surveyed contemporary national legislations, as well as scholarly articles addressing sustainable energy production and consumption. An analysis of the (inter)national jurisprudence was essential in drawing lessons to inform future climate litigation in South Africa. While a considerable number of researchers have written on the urgency for South Africa’s shift towards renewable energy sources, the approach taken in this paper place emphasis on the need to use collective action, through courtrooms and activism to stimulate political action towards sustainable energy production and consumption. This approach is key as there have been considerable documented accounts of the impact of rising emissions on youth, albeit fragmented.

## Results

Over the last half-century, some observers have commented on South Africa’s extreme climate events, with a mean annual temperature rising by more than 1.5 times the recorded global average of 0.65 °C [8]. Relatedly, the Intergovernmental Panel on Climate Change (IPCC) has projected with high confidence that by 2050, global temperature will increase from 1.8 to 4.0 °C [20]. This pattern is expected to worsen in light of the country’s emissions trajectory [8]. By emitting around 464 million metric tons (MtCO_2_e) of GHGs, it is ranked the highest emitter at the continental level [21]. As a consequence, (inter)national mean temperature will continue to rise above 4 °C over the twenty-first century [20]. The period implies that generation is an important determinant of one’s susceptibility to the impact of harmful emissions. For while the older generation who are mostly complicit in the emission will experience some of the effects of climate change, the young generation will bear the full brunt of hazardous gasses in subsequent years.

To be precise, youth are likely to inherit a planet 2.6 °C warmer, possibly populated by more than 11 billion human species, sea levels higher by 32 cm, and atmospheric concentration of approximately 623 parts per million by volume [9]. The variability is likely to trigger political contestation between citizens and the government as deluges, drought and extreme heat interfere with the enjoyment of basic rights, such as housing, food, and water securities [17]. This insecurity looms large especially in light of the ongoing Covid-19 crisis that has displaced many young people from their sources of livelihood, thereby worsening their ability to afford basic technologies that could enhance their adaptation to extreme heat.

It was in this light that the Paris Agreement entreats South Africa to take urgent action to mitigate high GHGs [14]. As a blueprint for conserving the environment for the young and succeeding generations, the Preamble specifically calls for intergenerational equity (IE) and moderation in the use of exhaustible resources. As a social concept, IE may be construed as a moral proclivity for fair consumption of resources to ensure the same resources will serve the needs of one’s progenies [22]. As an unwritten law, this moral code may somewhat be tied to the African identity and philosophy of ‘ubuntu’ [23]. Often captioned as ‘I am because we are’, one may loosely translate this idiom as ‘humanity towards others’ [23]. As a result, the notion of environmental conservation as entrenched under section 24(b) of the Constitution may well be said to have sprung from IE which is imbued in traditional customs. Drawing from its historical background, the constitutional provision underscores the need for fair consumption of natural resources without jeopardizing their future benefits.

At the international level, the notion of IE became the pivot around which hundreds of children sought to promote sustainability in the Philippines. In the celebrated *Oposa v Factorian*, the Supreme Court granted a petition by a group of minors and instructed the Secretary of the Department of Natural Resources to refrain from accepting, approving, or renewing timber license agreements [15]. In terms of IE, the Court observed that the class suit of the children, on the one hand, is to safeguard their right to a sound environment, and on the other hand, ensure the promotion of that right for the generations yet unborn. It concluded that whereas the right to a healthful environment is not under the Bill of Rights, it ‘concerns nothing less than self-preservation and self-perpetuation, the advancement of which may even be said to predate all governments and constitutions’ [15]. The judgment of the Court speaks directly to the rights of children, who are disproportionately impacted by the risks of a rapidly changing climate. This influential and powerful exposition of intergenerational rights in the *Oposa* case resonated in the judgment of the *Philippi Horticultural* case, where the High Court in South Africa held that development ought to be environmentally sustained for the benefit of present and future generations [24]. To this end, the universally ratified Convention on the Rights of the Child, and the African Charter on the Rights of the Child could be leveraged to advance IE and intergenerational climate justice.

Regardless of this creative application of IE, and the inclusion of some youth in the newly constituted Presidential Climate Change Coordinating Commission (P4C), there has been little progress towards a low-carbon economy and society. The inactivity of the Commission may somewhat be tied to its composition, as a greater percentage of its members are adults who are likely to suppress the novel suggestions of the few young members, including Ayakha Melithafa and Happy Khambule [25]. It is, therefore, unlikely that the country will radically shift from its over-reliance on coal in the next few years, thereby exacerbating the risks of youth to climate-related health risks, such as heat stress [28].

Broadly, heat stress may be read as excessive exposure of the body to heat which results in physiological or psychological impairment [26]. A narrow definition of the condition may be interpreted as an increase in the amount of heat beyond thermal comfort that negatively impacts the mental and physical condition of young people. Heat is an inevitable by-product of the human body. It is produced through a biochemical reaction and nerve coordination within the body. To sustain the proper functioning of the human system, the body occasionally discharges excess heat by circulating blood near the surface of the skin for cooling [26]. During seasons of an extremely hot climate, rooms or living spaces ought to be kept at a temperature below 24 °C during the night and 32 °C during the day [27]. As indicated in Table 1, under such temperate climates, heat is dissipated through three channels: evaporation of insensible perspiration (25%), convection in cool air (25%), and radiation (50%) [27]. Humidity plays a key role in the dissipation process, as it impacts the quantity of heat that can be distilled and evaporated [27].

**Table 1:** Dissipation of heat under temperate climate

Channels	%
Convection in cool air	25
Evaporation of insensible perspiration	25
Radiation	50

On average, the body’s core temperature fluctuates between a range of 36.1 and 37.2 °C [26]. When one’s temperature exceeds the threshold, all heat generated by the body must be dispelled through sweating. As a consequence, high humidity or excessive heat may result in slow air movement over the skin’s surface which could greatly inhibit the body’s cooling mechanism through sweating. The extremely hot climate has been perceived as impacting the psychological conditions of the young, increasing their vulnerability to violence, aggression, and irritability. Jointly, the (in)direct effect of climate change poses grave health risks for young people, which might span from depression, anxiety, post-traumatic stress, sleep disorders, and in an extreme case, suicide [26]. The health condition is further punctuated by fatigue, visual disturbances, tiredness, and dehydration [27]. Prolonged dehydration without replacement of the body fluid (through water intake) could lead to extreme medical conditions such as renal kidney, chronic kidney, heatstroke, and premature death [26].

Although high temperatures (in)directly impacts all young people, some are more prone to heat stress in light of their underlying health conditions or daily routine. Five sub-groups are pertinent in terms of vulnerability. First, young women. Some observers have long linked heat stress to violence among youth [28]. The psychological development of young people is often shaped by their daily experiences. When climate change alters the realities of these young people through extreme temperatures it (in)directly triggers frustration among young people which (in combination with existing patriarchal structures) might lead to aggression against vulnerable targets such as children and women [28]. As hot climate stimulates aggression, the onset of climate change might reinforce violent behavior among young people and towards others. Manifested by Cape Town’s ‘Day Zero’, severe drought and water scarcity placed enormous socio-economic hardship on residents [29]. Granted that the phenomenon spurred post-traumatic stress disorder (PTSD) in a small fragment of youth in the Western Cape, this translates into enormous mental health and social concern. The pattern is a further cause for concern in a country where one-sixth of its population have an underlying mental illness, including schizophrenia, bipolar disorder, anxiety, substance abuse, and depression [30]. In light of the spike in the number of gender-based violence (GBV), this development is worrying, not only because they might persist into adulthood, but might exacerbate GBV [31]. This raises concern for young people on campuses and in the (in)formal sector who might become victims of (accidental) attacks in light of increasing temperatures.

The second layer of youth susceptible to hot climate is young girls with obesity. In South Africa, 13.5% of young people are estimated to be overweight and obese (O/O) [32]. The burden of O/O induces heat stress, as body fatness determines one’s heat tolerance level. In a humid environment, aerobically fit youth are more capable of tolerating core temperatures of 0.9 °C higher before yielding to heat exhaustion. Table 2 highlights a cross-sectional study of 211 young people. As there is no available data for all genders, the table will be limited to only boys and girls. The survey found that whereas only 7.7% of boys were overweight, the same could not be said of girls as 21.1% were declared overweight [32]. Also, while only 1.0% of boys were obese, 4.6% of girls were seen as falling in the same bracket [32].

**Table 2:** Percentage of South Africa’s youth estimated to be overweight and obese

Gender	% Overweight	% Obese
Boys	7.7	1.0
Girls	21.1	4.6

Unlike their male counterparts, the susceptibility of young girls to heat stress is greater in light of their higher rates of O/O, which ultimately impacts their thermoregulation and heat tolerance [33]. Arguably, this situation provides some indication of why female voices have dominated the present-day climate movement, with Greta Thunberg, Licypriya Kangujam, and Alexandria Villaseñor being a few of the leading activists on the global scene [9]. Other reasons for the evolving gendered-climate activism may include: (i) inadequate democratic representation of women and other genders in political bodies; and (ii) lack of equality and occurrence of intersectional discrimination. As shown in Table 3, a similar pattern is visible at the local level, with schoolgirls Ayakha Melithafa, Yola Mgogwana, and Ruby Sampson distinguishing themselves as climate activists [10].

**Table 3:** List of climate activists

Name	Nationality
Greta Thunberg	Swedish
Licypriya Kangujam	Indian
Alexandria Villaseñor	American
Ayakha Melithafa	South African
Yola Mgogwana	South African
Ruby Sampson	South African
Happy Khambule	South African

Third, youth who spend a considerable portion of their day under direct exposure to the sun. Such young people include car park attendants, vendors, farm and factory workers. Although the young and older generations might share parallel thermo-regulatory systems, the former may be more vulnerable to heat stress in light of their intensive physical activity and insufficient rehydration. Unlike adults, youth spend a considerable portion of their days outdoors, thereby heightening their direct exposure to solar ultraviolet radiation (UVR). Constant exposure to extreme UVR from the sun(beds) could impair the genetic material (DNA) in normal skin cells, resulting in two effects: (i) acute (short) UVR exposure could result in tanning and sunburn; and (ii) chronic (repeated or prolonged) UVR exposure resulting in skin cancer and photo-aging [34]. These effects may be extended to other young outdoor workers, such as petrol attendants, forestry, and construction workers are equally susceptible as they are obliged to wear protective uniforms and pieces of equipment that might attract and retain heat.

Fourth, students in public schools. Air quality in classrooms is often shaped by the selection of masonry materials. Simply put, roofs and wall systems are critical in controlling CO_2_ circulation in enclosed spaces, such as classrooms [35]. Even in the dead of summer, a classroom constructed with cast concrete, concrete block, or brick could hold cool air inside for longer periods in light of their thermal mass-energy [35]. In any event, many public schools across the country are constructed out of corrugated iron roofs, remodeled shipping vessels, or asbestos sheeting [36]. These non-brick classrooms are characterized by higher indoor concentrations of CO_2_ as they provide a less spacious passageway for airflow. As summarized in Table 4, stale and stagnant air increases the risk of heat exhaustion and dizziness among students. As many of these educational institutions lack air conditions and a similar cooling system, one may argue that climate change might entrench and exacerbate existing poverty, as there could be poor performance in these schools. As an illustration, young learners in Johannesburg (comparatively mild climate zone) lamented fatigue, nausea, and dizziness when temperatures in the containers peaked beyond 47 °C [36]. Put a little differently, climate change might perpetuate inequality as extreme heat in overcrowded spaces impacts the academic performance of pupils from poor schools.

**Table 4:** Graphic impact of climate change

Demography	Possible impact
Asthmatics	Respiratory failure
Young men	Increased aggressive behavior
Students	Poor educational performance
Schools	Water insecurity
Youth working outdoor	Heat stress
Students in public schools	Dizziness and heat exhaustion
Asthmatics	Respiratory failure

Tied to the above group is the fifth, young asthmatics (see Table 4). The effect of climate conditions on airway reactivity is well documented. For persons with inflamed and narrow airways, the saturation of CO_2_ in classrooms or enclosed spaces obstructs air inhalation, with the possibility of causing respiratory failure [37]. Unlike dry climate which ameliorates the symptoms of asthma, the exposure of young asthmatic patients to poor indoor air quality or high humidity will frequently lead to acute exacerbations of their illness [37]. Pollen increments and dust generated through air pollution further compound the risks of allergies among such subgroup. Coupled with this setback is dehydration, in light of insufficient access to potable water to replenish the body’s fluid. With some schools dependent on rivers and boreholes, climate change might further threaten their water security on two fronts: (i) South Africa is a (semi)arid country which implies that prolong aridities might dry up river basins; and (ii) hurricanes and tidal waves could lead to contamination of water bodies such as streams with dangerous chemicals [38]. The latter is particularly disquieting as a disproportionate percentage of educational institutions and water bodies are situated near areas with industrial activities, mine landfills, and trafficked roads. When plagued with water insecurity, the conventional pattern of hand-washing will be jeopardized, especially in an era where the COVID-19 pandemic has made it mandatory.

In light of these threats, a discursive question that stares us in the face is, what legal safeguards are provided for young people from these hazards? At a basic level, a simple even though not simplistic answer to the question is that South Africa is obliged by the Paris Agreement to pursue sustainable development (SD) by mitigating GHGs [14]. As an evolutive concept, SD may be understood as the development that responds to the needs of the present generation without jeopardizing the prospects of future generations to utilize similar resources. Akin to the notion of IE and *ubuntu*, SD revolves around refraining from hazardous environmental activities that compromise the survival, sustenance, and wellbeing of the (future) population [39]. That cardinal principle conforms to section 7(2) of the 1996 Constitution that obliges the state to take all necessary measures to advance, fulfill and safeguard young people from any conduct which threatens the enjoyment of civil/political and social/economic rights of youth. That responsibility is expanded under section 24 of the document where the rights of youth to a clean environment are entrenched, and the duties of the state thereof. In recognizing the urgency for SD, section 24(b) obliges the state to avert pollution and ensure that the young generation inhabits an environment that is not harmful to their health and wellbeing. In simple terms, section 24 imposes a considerable obligation on the state to ensure that the young generation thrives in a safe environment. But what is the environment?

The term ‘environment’ undoubtedly conjures up thoughts of one’s physical surroundings, including the atmosphere, land, and water sources. These features might be seen as a narrow description of the (non-human) environment, and if one seeks to advance the rights of the young generation in this regard, then an overarching interpretation might be needed. Such clarification will encompass a consideration of the cultural, economic, and social interaction of man with the esthetic, chemical, and physical properties of the natural environment. In light of the country’s oppressive past and its vision for the future, this interpretation is appropriate as the environment does not merely shape one’s perception about life, but affects one’s health and mortality, particularly in the context of pollution. An almost identical position was adopted by Ngcobo J when he noted that economic development and ecological stresses are inexorably linked [40]. He further avers that since socio-economic development is vital for the realization of basic rights, any development which is detrimental to the environment ultimately impacts these rights [40]. To that end, section 24(b)(iii) highlights the inextricable link between ecological protection and human development when it underscores that the state ought to pursue 'ecologically sustainable development’ as a conduit for 'promoting justifiable economic and social development'. To a great extent, the Constitution could be said to be far-sighted when it drew from the Brundtland Commission and acknowledged SD, which was later rebranded as sustainable development goals nearly two decades later [41]. The notion of SD, as set out under section 24 of the Constitution, therefore, highlights the interrelationship between ecological conservation, social needs, and economic advancement.

Be that as it may, unlike a growing number of states where binding instruments have been adopted to curtail emissions, South Africa does not have such an instrument [42–44]. It is in this regard that the Department of Environment (DEA), on June 8, 2018, circulated a Climate Change Bill (CCB) for public comments [45]. A general overview of the White Paper indicates that it contains significant provisions that could fast-track GHGs mitigation. The Preamble of the Bill acknowledges the need to pursue environmental sustainability for the benefit of present and future generations. Section 1 proceeds to define climate change as natural climate variability as well as (in)direct human act which alters the compositions of the global atmosphere. Nonetheless, the major focus of the document is its aspiration to align policies and institutions, to provide comprehensive strategies for mitigation and adaptation. This is illustrated in section 8 where it provides an overarching architecture where national/provincial/municipal executives ought to collaborate in capping emissions and enhancing adaptation at local communities. Be that as it may, a critical reading of the instrument indicates that it follows the conventional practice of excluding youth from decision-making processes. Section 10(4)(e) of the instrument recognizes the participation of local communities, the private sector, and non-governmental organizations (NGOs) in framing adaption strategies. But, youth as a vulnerable constituency are omitted. It will be prudent for the National Assembly to reconsider this omission when the Bill is being debated on. The active involvement of youth co-designing coping strategies will play a key role in destigmatizing social biases, including the erroneous perception that outdoor schooling is degrading and inefficient. In contrast to this illusion, accessible green spaces help students learn better as they promote concentration, mental health, and environmental awareness. Still, since the contents of Bills are rarely altered in the National Assembly due to the relative dominance of the ruling party, young people will likely be excluded in the ensuing Climate Change Act [46].

In the absence of the Climate Change Act, the country’s efforts towards GHGs reduction could be assessed through the lens of two documents. First, NEMA: the prime objective of the instrument is the regulation of air quality. To be exact, section 24 of the instrument stipulates that any public or private entity that seeks to undertake any activity, including the construction of a new coal-fired power station, ought to secure an environmental authorization from the relevant actor. To this end, section 240(1) obliges the DEA to consider all relevant factors before granting authorization, including the pollution and environmental impacts of the proposed project. In this regard, an atmospheric emission license is granted to an applicant after the department has undertaken a thorough climate impact assessment of the project. The provision was put to the test in the *Earthlife* case where the NGO alleges that the department failed to undertake the required appraisal before authorizing the construction of a 1200-MW coal-fired power plant [47]. In its decision, the court upheld the claim of the applicant by emphasizing that the cardinal principles of NEMA were breached when the department failed to undertake a rigorous review of the proposed coal-fired power plant. To the court, the omission implies that the plant is likely to exacerbate emissions, extreme heat, and water scarcity [47]. It, therefore, instructed the department to carry out such an assessment [47]. The judgment illustrates that although there might not be a definite Climate Change Act, there is an explicit legal obligation within section 38 that binds the state to ensure that the energy sector does not emit GHGs that threaten life and health. Nonetheless, unlike Climate Change Act, the instrument departs from strict emission reduction. To be specific, it does not provide stringent measures on how the energy sector could reach net-zero or carbon-negative in the coming years.

The second instrument that seems to provide a specific response to GHGs is the Carbon Tax Act (CTA) [48]. Gazetted on 1 June 2019, the instrument is hinged on the ‘polluter pays’ notion where those culpable of atmospheric depletion ought to bear the cost. As a carbon-pricing mechanism, the primary focus of the instrument is the private sector, mainly industries, and individuals. Three forms of emissions are taxable: those from (i) industrial processes; (ii) fugitive emissions; and (iii) fuel combustion [48]. The Act is operationalized in two phases. According to section 5 of the Act, the first phase spans from 1 June 2019 to 31 December 2022. Within this duration, levies are imposed on direct (scope 1) emitters, which include companies and industries which directly burn coal. With an initial tax rate of R120 (approx. $8) per tone of CO_2_ equivalent of the GHGs of the company, the levy can be cut down to up to 95% following a trend of performance incentives, allowances, and tax breaks [48]. Since the imposition of the tax, there have been considerable hikes in fuel prices, with diesel increasing by 10c/l and petrol by 9c/l [49, 50]. Ultimately, these price increases have trickled down to young consumers as food and basic commodities are affected by hikes in transport [51]. The second phase of CTA is scheduled to commence in 2023. This period is projected to witness an increase in revenue collection. At this stage, the rate of R120 is projected to escalate annually by the consumer price inflation plus 2% of the standard rate [48].

Akin to NEMA, CTA falls short on three grounds: (i) the substantive nature of the levy. In its entirety, the fine could be argued as inefficient towards a radical shift to renewables or fewer emissions. Perhaps, insertion of a tax-free benchmark could have encouraged one not to exceed such a standard instead of incurring fines; (ii) failure to set out clear guidelines on how to use the proceeds. As it stands now, there is a lack of clarity on how proceeds will be dispensed. This obscurity is more glaring as the Act takes a step back from ring-fencing the revenues for exact climate-related intervention. This contention has the potential of opening a flood gate for critics to assert that perhaps the mechanism is the latest government money-spinning machinery to recoup revenue for its depleted coffers. It might be useful to perhaps insert a provision in the Act indicating that the proceeds will be recycled into (re)afforestation, renewables for poor communities, and social infrastructures to moderate the negative effect of GHGs on youth. (iii) The third layer is the exemption of the energy sector from similar fines. The energy sector contributes 84% of the approximate 464 million metric tons (MtCO_2_e) GHGs emitted by the country [3]. Of this figure, two state-owned entities (SoEs), Eskom and Sasol account for 42% and 11%, respectively [50]. Sasol depends on fossil fuels such as coal for the production of steam, electricity as well as gasification feedstock [50]. Eskom, on the other hand, relies on three coal-fired power plants in Medupi, Kusile, and Kendal to generate approximately 90% of the country’s electricity [52]. This consumption results in the use of more than 90 million tons of coal per annum, thus reversing attempts towards GHGs mitigation [52].

With urbanization and the rising electricity demand, coal consumption is expected to increase rather than decrease. Perhaps this provides the rationale for the recent diversification of the operations of Alexkor (SOE) into fossil fuel exploration. As indicated on the entity’s website, the diversification is to ensure a continuous supply of 60-million tons of coal needed by Eskom per year [52]. This high consumption ultimately makes the country’s energy sector the most coal-dependent of the G20 countries [52]. As a consequence, although the energy sector makes a significant contribution to economic advancement, its high coal consumption poses a considerable threat to the environment and gravely interferes with the rights to life, health, and survival of youth. Begging the question, what action is suitable for capping state-owned entities’ (SOEs) GHGs?

In recent times, one of the evolving strategies used by young people to hold their states accountable for climate inaction has been climate change litigation (CCL). Aside from collective acts such as rallies, sit-ins, and strikes, some youth continue to exercise their rights by filing cases against (inter)national governments for lack of political will towards GHGs. At the minimum, this evolving and radical field is used by a growing number of citizens to hold states and third parties liable for harms suffered in light of rising temperatures. Drawing from evolving CCL jurisprudence, the next section assesses why young South Africans have arguably not undertaken similar climate litigation, and whether they have the locus stand to pursue such a cause.

## Discussion

Youth were the main drivers of the anti-apartheid campaign in South Africa. However, it appears a new form of apartheid has emerged. Climate change, in the form of extreme heat and water scarcity, seems to undermine the fundamental freedoms to life, health, and wellbeing of this subpopulation. To safeguard the environment for future use young people ought to actively participate in mitigation efforts, mainly through climate litigation. Definition of climate litigation abound. It has been proffered as a legal action that concerns environmental issues. Another observer decodes it as an application inspired by climate concerns or to mitigate GHGs. In merging all these different constructions, the present paper will construe climate litigation as any petition which expressly or implicitly raises issues of GHGs and their effects. This expansive definition embraces legal actions that allege overt environmental pollution, ineffective environmental impact assessment, non-compliance with constitutional obligations, and continuous fossil fuel exploration. CCL could be said to achieve two simple objectives: provide remedies and forestall future violations. In other words, the ultimate aspiration of this action is to minimize or at most halt the potential effect of GHGs.

Of the many CCL, two cases are worth citing. The first relates to the UN Committee on the Rights of the Child petition whereby 16 young people argue that the failure of states to mitigate emissions constitutes an infringement of their rights [17]. Second, having witnessed extreme heat amidst wildfires, six Portuguese young people aged 8–21 applied to the European Court of Human Rights, alleging that the failure of 33 European countries to effectively cap their emissions infringes on their rights to life and livelihoods [53]. Similar sentiment has been expressed across the UN, Canada, Columbia, and the Netherlands, where youth have filed complaints against their respective governments [15–18].

### Conceptual constraints

Despite the rich climate litigation jurisprudence, and facing similar risks, young South Africans have not presented a similar petition to the Constitutional Court for consideration. This paradox may be linked to five conceptual and practical constraints. At the conceptual level, there has been an ancient contestation around the competence of local courts to adjudicate on socio-economic issues, including environmental complaints. In contrast to the conventional practice of judges assessing cases of affected parties listed on a petition, the issue of climate change transcends one specific community. One might argue that courts lack the necessary expertise to adjudicate on environmental matters, as the causation and impact of global warming involves multiple players at the (inter)international levels. To that end, the energy sector (as respondents) might propose political intervention rather than legal scrutiny. Notwithstanding, considering that there is a widely perceived lack of political action to coal consumption, and the National Assembly is dominated by the ruling party, it is unlikely that parliamentary could provide a radical panacea towards renewables [38]. Suffice it to note, however, that the obstacle is not insurmountable as local courts have adopted an activist approach towards human rights more broadly, and environmental justice in particular [47].

The second conceptual issue young litigators might have to contend with is providing airtight arguments suggesting that present-day heatwaves or harms suffered are due to the actions of the state’s energy sector. A basic argument could be that the energy sector consumes an enormous amount of coal that emits vast MtCO_2_e of GHGs which merge with other gasses in the atmosphere to increase warming. At this stage, one needs to depart from mere speculation and rather rely on existing data to buttress fears of climate-induced disasters, such as drought and heat stress. Granted that the court is amenable to global development, then the reports and projections of the IPCC could be a useful starting point. Expert testimony, in the form of climate modelers and scientific reports, could be used to substantiate the link between emissions and warming. Young people continue to be victims of water shortages and heatwaves. This observation may be extended to encompass the lack of proactive measures from the state to avert the onset of drought in the first place, and/or potential water shortages. With drought serving as an underlying factor of water shortages, one may tie youth’s water insecurity to the energy sector’s fossil fuel consumption. Yet it remains contentious to directly tie the energy sector to climate change as current emissions do not trigger a completely new phenomenon, but rather exacerbate existing climatic conditions. This is more the case as emissions could have extended life-span in the atmosphere, making it difficult to determine which emission triggered a particular heatwave.

In the face of these complexities, a clear-cut argument perhaps might be that a substantial quantity of the energy sector’s emissions significantly contributed to atmospheric depletion and resultant hot temperatures. Drawing from contemporary jurisprudence, a cardinal principle that could be invoked to advance CCL is the age-old notion of public trust [54]. As a custodian of natural resources, the state must utilize resources in a manner that is reasonable and promotes the welfare of its general population. This concept was the crux of an argument made by some young people who claim that the failure of the government to adopt and operationalize climate recovery policy constitutes a breach of its responsibility to safeguard vital resources and their rights to the property [54].

Also, the public nuisance claim provides an important entry point for holding the state accountable [55]. The principle implies that the state is complicit in an obstructive act that interferes with the rights of the general public. A similar assertion was articulated in *Leghari v Pakistan*, where a local farmer filed a petition alleging that the government’s insufficient climate action violated basic rights [55]. In upholding the claim, judge Ali Shah noted that indeed rising GHGs symbolized present-day threat to mankind, and the state must take urgent measures to fast-track the operationalization of its national climate policy. To that end, the energy sector’s emissions qualify as a public nuisance as it impinges on a sizeable number of the population. This is particularly the case as access to water and good health are entrenched rights that the state ought to safeguard, rather than interfere with their enjoyments.

The next important element to consider is the procedural and substantive nature of potential climate litigation. At the onset, an important question that stares us in the face is whether local youth have a locus standi to file a climate suit. One of the principles embedded in section 34 of the Constitution is that victims of abuse have the right to approach a competent court to have their abuse halted or averted. The provision specifically avers that ‘[e]veryone has the right to have any dispute that can be resolved by the application of law decided in a fair public hearing before a court or, where appropriate, another independent and impartial tribunal or forum.’ To Ngcukaitobi, such an aggrieved person ought to illustrate that: (i) s/he has suffered an injury; (ii) there is an identifiable perpetrator; and (iii) a court decision could remedy the injustice [56]. Indeed, South Africa’s continuous reliance on coal constitutes a prima facie breach of sections 7 and 24 of the Constitution. The constitutional violation in itself constitutes a clear locus standi for young people to file similar petitions. The drought, ongoing heatwaves, and water scarcity provide sufficient grounds for young people in satisfying the three thresholds.

### Practical constraints

Despite this optimism, four practical barriers might threaten young people’s pursuit of climate justice through courtrooms. One of the primary deterrents could be the enormous financial resources of the energy sector. The pool of financial and technical resources that Eskom and Sasol command could be used to deter CCL at three levels: (i) delay the proceedings; (ii) buy-out leads plaintiffs; or (iii) hire experts to quash the case based on general causation of high temperature. The likelihood of operationalizing any of these strategies could serve as a red flag for young activists who might have considered the arduous task of CCL.

Second, insufficient resources have perhaps undercut attempts towards mobilization. Ideally, while all youth ought to collectively submit a CCL, not all have the capacity and technical know to undertake this assignment. Although have-nots are more susceptible, they might lack the financial resources to challenge emitters. As a result, one of the important features of recent youth CCL has been the level of collaboration with NGOs. One such organization that has been visible is Our Children’s Trust. The Trust has been a notable advocate for climate mitigation by supporting landmark cases that seek to forestall fossil fuel exploration in the US. In their attempt to file similar landmark cases, young South Africans need to build a similar partnership with philanthropic funders who may provide the necessary financial and technical assistance.

Third, the prolonged and time-consuming nature of litigation. A considerable number of CCL has been marked not merely by the volume of resources committed, but the frustration litigants endure in light of undue delays in handing down decisions. The arduous task of prolonged judgment has perhaps served as a disincentive for young people seeking to undertake CCL.

Fourth, there is a problem of insufficient awareness around rising emissions. Climate activism, to a greater degree, is nascent in many localities. This setback is compounded by the lack of awareness of the impact of GHGs and their effects on humanity. As corporations use (print/electronic) media to disabuse public fears about climate change, attempts to mobilize might be inhabited by young people’s imperfect knowledge of GHGs. This hindrance may be tied to a breach of consumers’ right to know by South Africa’s energy sector. As a simple rule of thumb, producers ought to inform their consumers of the anticipatory risks which a product might incur, particularly those which impact human health and the environment. In line with this principle, section 3(1)(e) of the Consumer Protection Act (CPA) oblige producers and distributors of any services to provide adequate information and raise the awareness of consumers about the content of their products. Section 61(1)(c) adds a caveat to the above provision when it stipulates that a producer or retailer may be liable for any harm accruing from ‘inadequate instructions or warnings’ provided to the consumer about any hazard arising from or associated with the use of any goods or services. As a consequence, the alcohol and tobacco industries frequently warn their consumers about the hazards of their products. Even so, the same cannot be said of Eskom and Sasol although consumption of electricity and fuel adversely impact the environment and human health. In essence, by withholding such crucial information from consumers, it breaches section 32(b) of the Constitution, relating to the rights to key ‘information that is […] required for the exercise or protection of any rights’. Of course, insufficient awareness among consumers is worrying as the energy sector is arguably aware of the impact of their operations and possible effects on end-users, such as young people. By being privy to the dangers of GHGs from their operations, a fraction of the population might shift to solar panels, or energy-efficient transport systems such as bicycles or public transport. By extension, this approach could have mitigated the number of emissions generated by these two SOEs. Accordingly, litigation around these provisions could serve two purposes: (i) hold the energy sector accountable for withholding key information from its consumers; (ii) trigger an institutional action towards raising awareness on the impact of GHGs, and (iii) use the legal action itself to draw the public attention to climate change.

Having said that, it would be impracticable for courts to completely halt coal consumption, as that would bring operations of the energy sector to a halt. As vanguards for citizens’ rights, courts may perhaps contribute to GHGs mitigation through three channels: (i) injunctive relief; (ii) award compensation; or (iii) both. Injunctions may be sought to halt or enjoin the state to cap its GHGs as continuous reliance on coal is unreasonable in the context of climate change. As illustrated in *Pandey v Union of India*, compensation in this context may constitute instructing the state to adopt the Climate Change Act, and effectively operationalizing its provisions to reduce SoEs emissions [57]. In filling a climate justice complaint, young South Africans could make three submissions.

In the first place, the reduction of coal consumption by 55% by 2030. This projection is drawn from the *Neubauer and others v Germany* ruling, where the country’s highest court, the Karlsruhe instructed the state to limit by 55% the greenhouse gas emissions by 2030 [58]. The judges ordered a reduction of emissions within the agriculture, construction, energy, and transport sectors. It is anticipated that a reduction in coal consumption by South Africa’s energy sector might lead to a gradual phasing-in of renewables within this time frame. Although some policymakers might be sceptical of its feasibility, the recommendation is not farfetched as a tighter deadline was imposed by the Dutch Supreme Court where it directed the state to slash its GHGs emission by 25% within a year [18]. This claim presents better prospects of obtaining judicial approval, as well as executive compliance as it provides a reasonable time frame for operationalizing it. Second, provision of potable water in public spaces to mitigate dehydration, and construction of rainwater collectors in townships to improve young people’s access to water. Third, the establishment of an early warning system (EWS). The urgency of EWS is to detect the resurgence of heatwaves to provide practical guidelines on how youth ought to adapt. It was against this backdrop that an Indian court instructed the government to undertake remedial action to avert climate risks in the Himalayas [59]. In anticipation of hot temperatures, the state could use social media to forewarn young people on the need to observe basic protocols such as wearing a loose cotton dress, sufficient water intake, frequent breaks, and shorter class hours. This effort could be complemented with frequent testing of young people to detect whether some have experienced heat stress. Early detection of heat stress has the potential of ensuring timely recovery of victims to ensure the risk does not exacerbate into a serious complication.

Yet, as a reasonably cautious entity, and considering the lengthy period of litigation, it will be worthwhile for policymakers to undertake six urgent actions to safeguard the health of its young population. First, improve natural ventilation through (re)afforestation on campuses and urban environments to provide cooling and shade during summer. This intervention is essential as a conducive climate has the prospect of enhancing the cooling mechanism of the body, thereby lowering heat-related health concerns, such as dehydration, asthma, and heat stress among the young population. Second, provision of tap water on vantage points for easy access by youth. Third, this effort could be complemented with adapting school and work hours to hot summers, with students, miners, and farmworkers granted longer breaks. This implies that they may commence early and close late. Fourth, the replacement of prefabricated classrooms with concrete structures. In cases where this recommendation seems difficult to operationalize, the installation of air condition or cooling gadgets in these classrooms could boost thermal comfort. Fifth, the construction of leisure centers on campuses. These venues will be conducive to providing cool air for students who might utilize it during extremely hot days. To enhance thermal comfort, this room may be surrounded by trees, with internal features composed of wall plants, a white roof system, and wall fans. Considering the amiability that this room might provide, it could be utilized as an examination center to enhance critical thinking during hot seasons. To ensure equity, students could be randomized. Finally, intensify awareness campaigns around climate change, and the need for the population to shift to energy-efficient transport systems and/or solar water heaters for households.

## Conclusion

The South African Constitution imposes an obligation on judges to ensure that the state or third parties refrain from activities which (might) violate the rights of citizens. As a result, courts have adopted an activist approach to the Constitution in their attempts to foster environmental rights in particular, and human rights more broadly [60]. This is more the case as the emissions of SoEs, such as Eskom and Sasol have contributed to global warming, resulting in high temperatures and drought among many localities. Yet, although this phenomenon persists, amidst a surging global movement of youth submitting CCL, young South Africans have rarely used courtrooms to cap emissions.

The paper argues that this irony might be linked to the lack of capacity to undertake this action, or insufficient information on the adverse effect of emissions on their health. The paper observes that considering the government’s perceived slow steps towards shifting from coal to renewables, youth (who will bear the brunt of high emissions) ought to submit similar CCL to the local court for consideration. Their application may be underpinned by (i) sections 7 and 24 of the Constitution where the rights of youth to health and sustainable development are, respectively, entrenched; (ii) sections 61 of the CPA which oblige the energy sector to raise awareness of the side-effect of their product.

In pursuing this course, the group: (i) must illustrate that they have suffered extreme hardship in light of SoE’s emissions; (ii) partner with an NGO in light of the enormous resources needed for CCL; (iii) make a reasonable plea of capping emissions by 25% in a year; (iv) intensify climate awareness and shift to energy-efficient transport systems. It is anticipated that these measures will make a significant contribution towards shifting the country to a low-carbon economy.

## Data Availability

Not applicable.

## Abbreviations

CCB: Climate Change Bill
CCB: Climate Change Bill
CCL: Climate change litigation
CPA: Consumer Protection Act
CTA: Carbon Tax Act
DEA: Department of Environmental
EWS: Early warning system
GBV: Gender-based violence
GHGs: Greenhouse gas emissions
IE: Intergenerational equity
IPCC: Intergovernmental Panel on Climate Change
MtCO_2_e: Million metric tons
NEMA: National Environment Management: Air Quality Act
NGOs: Non-governmental organizations
O/O: Overweight and obese
P4C: Presidential Climate Change Coordinating Commission
PTSD: Post-traumatic stress disorder
SD: Sustainable development
SOEs: State-owned entities
UVR: Solar ultraviolet radiation
